# Single-Cone vs. Carrier-Based Root Canal Obturation with a Calcium-Silicate-Based Sealer: An In Vitro µ-CT Analysis

**DOI:** 10.3390/biomimetics11020152

**Published:** 2026-02-19

**Authors:** Vincenzo Tosco, Riccardo Monterubbianesi, Michele Furlani, Andrea Spinelli, Fausto Zamparini, Giovanna Orsini

**Affiliations:** 1 Department of Life Sciences, Health and Health Professions, Link Campus University, 00165 Rome, Italy; v.tosco@unilink.it (V.T.); m.furlani@unilink.it (M.F.); 2 Department of Clinical Sciences and Stomatology, Università Politecnica delle Marche, 60126 Ancona, Italy; g.orsini@univpm.it; 3 Endodontic Clinical Section, Dental School, Department of Biomedical and Neuromotor Sciences, University of Bologna, 40125 Bologna, Italy; andrea.spinelli4@unibo.it (A.S.); fausto.zamparini2@unibo.it (F.Z.)

**Keywords:** endodontics, root canal obturation, calcium-silicate-based sealers, micro-computed tomography, carrier-based obturation, single-cone technique

## Abstract

The introduction of calcium-silicate-based sealers has renewed interest in simplified obturation protocols such as the single-cone technique, although warm techniques, including carrier-based obturation, are still considered the gold standard. The aim of this in vitro study was to compare the quality of root canal obturation achieved with single-cone and carrier-based techniques when used with the same calcium-silicate-based sealer. Thirty extracted mandibular molars were prepared using a standardized rotary instrumentation protocol and randomly assigned to two groups (n = 15 each): Group A was obturated using a carrier-based technique (Soft-Core obturators), while Group B was obturated with the single-cone technique. All canals were filled with the same calcium-silicate-based sealer (NeoSEALER Flo). Micro–computed tomography was used to evaluate the number and volume of voids of the obturation. Quantitative analysis showed that Group A exhibited a significantly lower number of voids (9.0 ± 5.0) and reduced total void volume (2.58 ± 0.8 mm^3^) compared with Group B (22.0 ± 10.1 voids; 4.71 ± 1.1 mm^3^; *p* = 0.00002 and *p* = 0.0026, respectively). Qualitative analysis confirmed that carrier-based obturation achieved a denser and more homogeneous filling, while the single-cone technique showed larger voids mainly in the coronal and middle thirds. Both techniques provided a reliable apical seal. Within the limitations of this in vitro study, carrier-based obturation demonstrated superior overall filling quality compared with the single-cone technique when used with a calcium-silicate-based sealer, particularly in the middle and coronal regions of the root canal.

## 1. Introduction

The long-term success of endodontic therapy relies on the thorough elimination of infection and the three-dimensional sealing of the root canal system. Despite advances in instrumentation and irrigation, the quality of obturation remains a critical determinant of prognosis. Inadequate sealing, particularly at the apical third, is strongly associated with bacterial reinfection and the persistence of periapical lesions [1,2]. Clinical data suggest that a substantial proportion of endodontic failures are directly linked to incomplete or defective root canal filling [3].

While cold lateral condensation represents the traditional obturation technique, warm vertical compaction has been extensively described as a benchmark method for achieving three-dimensional filling of the root canal system due to its improved adaptation and homogeneity [4,5]. Its ability to plasticize gutta-percha allows dense fillings and better adaptation to irregularities, isthmuses, and lateral canals [6]. However, this technique presents some limitations, including shrinkage of gutta-percha upon cooling, technical sensitivity, and the potential for sealer extrusion [7]. These drawbacks, combined with the increasing demand for simplified protocols, have driven research into alternative approaches.

The introduction of calcium-silicate-based sealers (CSSs) has significantly changed the clinical scenario [8]. These materials, often vaguely referred to as bioceramic sealers [9], are biocompatible, bioactive, dimensionally stable, and capable of setting in the presence of moisture and blood [10]. Their bioactivity promotes the release of calcium ions and the nucleation of apatite, potentially improving the sealer–dentin interface and supporting periapical healing [11]. These properties support the use of the single-cone (SC) technique, where gutta-percha functions mainly as a carrier for the sealer. The SC approach simplifies the clinical procedure, is less operator-dependent, and reduces the risk of heat-related stress to periodontal tissues [12,13]. Nevertheless, concerns remain regarding excessive reliance on sealer, difficulties in retreatment, void formation and the risk of underfilling [14].

Recently, new formulations of CSS have been designed to tolerate moderate heat, enabling their use not only with cold SC obturation but also in combination with thermoplasticized and carrier-based techniques [15]. Warm vertical compaction combined with CSS may offer greater adaptation in complex anatomies [16], yet the potential risk of shrinkage and extrusion persists. Conversely, SC obturation simplifies the procedure and reduces operator variability but may increase the risk of sealer-rich fillings with interfacial voids [17,18]. NeoSealer Flo is a premixed CSS that sets through a moisture-dependent hydration reaction. Its formulation, which includes calcium silicate and calcium aluminate phases combined with radiopacifiers, confers high flowability and a hydraulic setting mechanism [14]. These physicochemical properties may influence sealer thickness, spatial distribution, and void formation within the root canal system, depending on the obturation technique applied.

Several recent studies, including systematic reviews, have compared these techniques; however, results remain inconsistent [19,20,21]. Some investigations report comparable healing and sealing outcomes between SC and traditional warm obturations techniques [17,22], whereas others emphasize higher apical gaps or increased voids when the SC method is used [13,18]. Moreover, most available evidence evaluates different sealers across techniques, leaving limited data on the direct comparison of the same CSS applied with both approaches and rarely investigating the newer formulations specifically designed to tolerate heat.

Therefore, the present study aimed to evaluate and compare the quality and quantity of obturation using a CSS applied with the SC technique and with the warm carrier-based (WCB) technique, analyzed through high-resolution micro-computed tomography (µ-CT). The null hypothesis was that there would be no significant differences in obturation quality, assessed by three-dimensional void distribution and volume, between the two obturation techniques when using a last-generation CSS.

## 2. Materials and Methods

### 2.1. Specimen Collection

Thirty extracted human mandibular molars were selected from a pool of teeth removed for periodontal or orthodontic reasons from subjects aged between 18 and 30 years. According to the Local Ethic Committee guidelines and WMA Declaration of Helsinki (2013) [23], informed consent was obtained from the subjects that were aware that their hard-dental tissues, as a byproduct of the surgical procedures would be used for research purposes. Inclusion criteria were intact coronal surfaces, absence of caries, two separate roots with two mesial canals and one distal canal with fully formed apices, and a root angulation between 10° and 30°. Exclusion criteria, including root resorption, immature apices, fractures, previous root canal fillings, and significant calcifications, were detected using a ×10 objective (Eclipse Ni, Nikon, Amstelveen, The Netherlands). Mandibular molars were selected to provide a standardized and anatomically complex root canal system, allowing a reliable evaluation of obturation quality. In selected teeth, remnants and connective tissue debris were removed with manual and ultrasonic scaling. Therefore, the teeth were decoronated to obtain a standardized root length of approximately 15 mm, which was used as a reference to define three virtual regions of interest (coronal, middle, and apical thirds), each measuring 5 mm, for micro-computed tomography (µ-CT) analysis. Samples were stored throughout the study in 0.5% (wt/wt) chloramine (NH_2_Cl) solution at room temperature (27 °C), which was refreshed every two weeks.

Each tooth was scanned with µ-CT both before and after root canal obturation in order to establish baseline anatomical parameters and to allow a precise comparative evaluation of voids. Indeed, based on root canal system volume and comparable anatomical features, samples with similar morphology were selected for inclusion in the study, ensuring uniformity and minimizing potential bias related to anatomical variability. Scans were performed using a Bruker SkyScan 1174 system (Skyscan, Kartuizersweg, Kontich, Antwerp, Belgium) installed at the Laboratories of the Center for Nanostructure Microscopy Research and Service (CISMiN), Università Politecnica delle Marche. Acquisition parameters were standardized and conserved identical for both pre- and post-obturation scans: 50 kV X-ray source voltage, 800 µA current, and a 1 mm aluminum filter to minimize beam hardening. Pixel size was set to 11.5 µm, with 0.4° rotational steps over 180°, and an exposure time of 10 s per projection. To cover the entire specimen in the z-axis, two consecutive acquisitions were performed for each tooth. The average scanning time was approximately 4 h per sample, yielding about 950 projections.

Reconstruction of cross-sectional images was performed with NRecon software (version 1.6.10.2, Bruker, Billerica, MA, USA), applying consistent correction parameters for all acquisitions: ring artifact reduction (4.0), smoothing (5.0), beam hardening correction (35%), and individualized misalignment compensation. The reconstructed datasets were then analyzed using Dragonfly 2022.2 software (Comet Technologies Canada Inc., Montreal, QC, Canada) to perform three-dimensional (3D) evaluations, ensuring that identical measurement protocols were applied to pre- and post-obturation scans for accurate quantification of filling material and voids [24].

### 2.2. Root Canal Instrumentation and Obturation

All root canal preparation and filling procedures were carried out by a single operator, an endodontic specialist, to ensure standardization. After access cavity preparation, canal patency was confirmed using a size 10 K file (Zarc4Endo, Gijón, Spain), and the working length was established 0.5 mm shorter than the apical foramen using an electronic apex locator (Root ZX, Morita Europe, Dietzenbach, Germany) [17,25]. A glide path was prepared up to a size 15 K file.

Instrumentation was performed with BlueShaper Pro rotary files (Zarc4Endo, Gijón, Spain) in the sequence recommended by the manufacturer (Z1, Z2, Z3, and Z4) at 500 rpm and 4 Ncm torque. Irrigation was carried out at each step of instrumentation for 1 min with 2.5% sodium hypochlorite and 17% EDTA, using an IrriFlex 30-gauge needle (Produits Dentaires SA, Veyvey, Switzerland) introduced 2 mm short of the WL [26]. At the end of instrumentation, the canals received a final irrigation with the same protocol and were dried with paper points calibrated with the last shaping file (Z4).

After instrumentation, specimens were randomly assigned into two experimental groups according to the obturation technique:

Group A (n = 15): WCB obturation with Soft-Core obturator (CMS Dental, Glyngore, Roslev, Denmark) in combination with CSS NeoSEALER Flo (Avalon Biomed, NuSmile, Ltd., Houston, TX, USA). Soft-Core obturators were selected according to verifiers. NeoSEALER Flo was placed on the canal walls. Obturators were heated in a Soft-Core oven (CMS Dental) following manufacturer instructions and then inserted slowly to full working length. Excess material protruding coronally was removed with a Therma-Cut bur (Maillefer Dentsply Sirona, Charlotte, NC, USA).

Group B (n = 15): SC obturation with a gutta-percha master cone coated with the CSS NeoSEALER Flo (Avalon Biomed, NuSmile, Ltd., Houston, TX, USA). Calibrated gutta-percha master cone (Z4 Gutta-percha points, Zarc4Endo) was used. NeoSEALER Flo was delivered into the coronal half of the canal using a premixed syringe with a 25-gauge disposable needle (NeoSEALER Flo tips, Avalon Biomed, NuSmile, Ltd., Houston, TX, USA). The master cone was coated with sealer and inserted to full working length without pumping or rotation. Excess gutta-percha was removed at the canal orifice with a heated plugger.

After obturation, periapical radiographs were taken immediately to verify the quality of the filling procedures. Subsequently, all specimens were stored at 37 °C and 100% humidity for 14 days to allow complete setting of CSS, in accordance with previous µ-CT studies [27,28]. Prior to micro-CT scanning, specimens were anonymized and assigned a coded identification number to ensure blinding. Specimens were then rescanned using the same micro-CT parameters described above. The reconstructed datasets were analyzed with VG Studio MAX (Volume Graphics, vers.1.2.1, Heidelberg, Germany) to calculate the number and volume of detected voids (mm^3^).

Three-dimensional void detection and quantification were performed by a single trained investigator, blinded to the obturation technique, using an automated segmentation and morphometric analysis workflow. In the three-dimensional reconstructions, voids and filling materials were highlighted in different colors for improved visualization. In the present study, obturation quality was assessed by three-dimensional void analysis. In µ-CT evaluation, interfacial gaps and apical sealing ability are indirectly represented by the presence, distribution, and volume of voids rather than by separate linear measurements. For void detection, a minimum detectable void volume (MDVV) was defined based on the spatial resolution of the µ-CT system (voxel size: 11.5 µm). Objects smaller than 27 voxels (3 × 3 × 3) were excluded to minimize image noise, corresponding to an MDVV of 4.1 × 10^−5^ mm^3^. For descriptive purposes only, voids were further categorized according to their volumetric extent as small, medium, or large, as detailed in Supplementary Table S1.

### 2.3. Statistical Analysis

The statistical analysis of the morphometric data obtained from the μ-CT was performed using Prism 9 (GraphPad Software, Inc., San Diego, CA, USA). The normality of the data distribution was assessed with the Shapiro–Wilk test (*p* > 0.05), and the homogeneity of variances with Levene’s test (*p* > 0.05). Although the number of voids represents a count variable, it was treated as a continuous outcome because values were sufficiently large and approximately normally distributed, allowing parametric comparison of group means. Since both assumptions were satisfied, comparisons between Group A and Group B were carried out using independent-samples *t*-tests on the following parameters: number of voids and void volume (mm^3^). All data were expressed as mean ± standard deviation (SD). Statistical significance was set at *p* < 0.05. The sample size for each group was determined a priori using G*Power (version 3.1.9.7, Heinrich-Heine-Universität, Düsseldorf, Germany) to achieve a statistical power of 0.80 at a significance level of 0.05, which required a minimum of 13 samples per group. To account for possible specimen loss, 15 samples were included in each group.

## 3. Results

**Figure 1:**
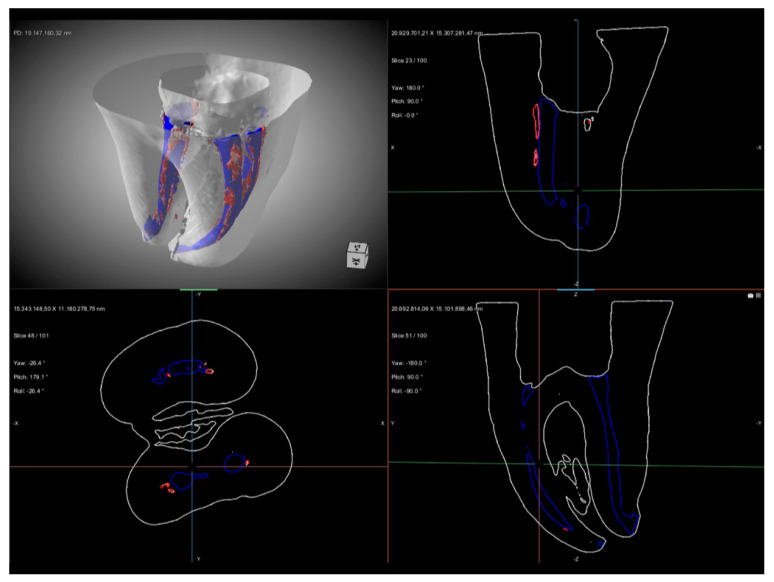
Representative µ-CT images of Group A (carrier-based obturation with calcium-silicate-based sealer). The figure shows a 3D rendering together with multiplanar axial and sagittal sections of the root canal system. The obturated material is displayed in blue, while voids are highlighted in red. Voids were mainly observed in the coronal and middle thirds, while no voids were detected in the apical third. The apex appeared completely sealed without detectable voids.

**Figure 2:**
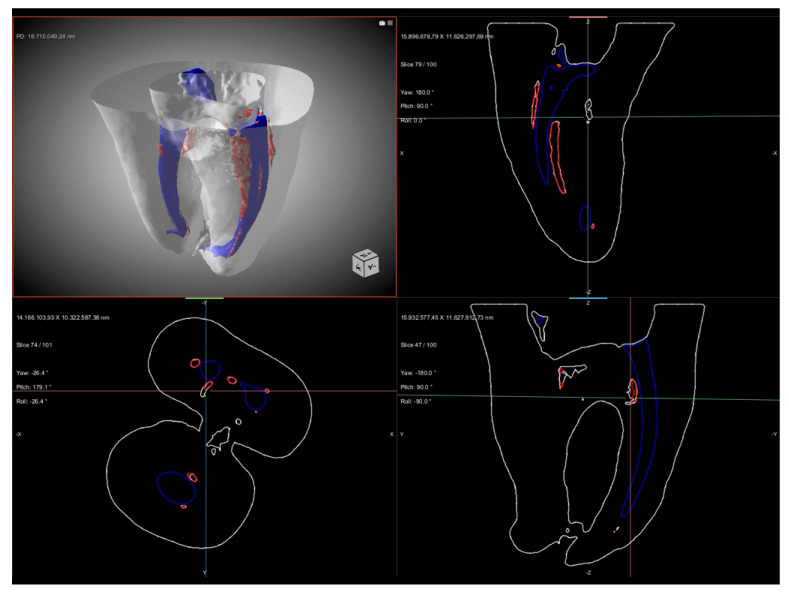
Representative µ-CT images of a Group B (single-cone obturation with calcium-silicate-based sealer). The figure shows a 3D rendering together with multiplanar axial and sagittal sections of the root canal system. The obturated material is displayed in blue, while voids are highlighted in red. Larger voids were predominantly located in the coronal and middle thirds, with additional voids also observed in the apical third. The apex appeared completely sealed without detectable voids.

µ-CT analysis allowed both a qualitative and quantitative evaluation of the obturation quality obtained with the two techniques. Representative 3D reconstructions of Group A and Group B are shown in Figure 1 and Figure 2, respectively.

**Figure 3:**
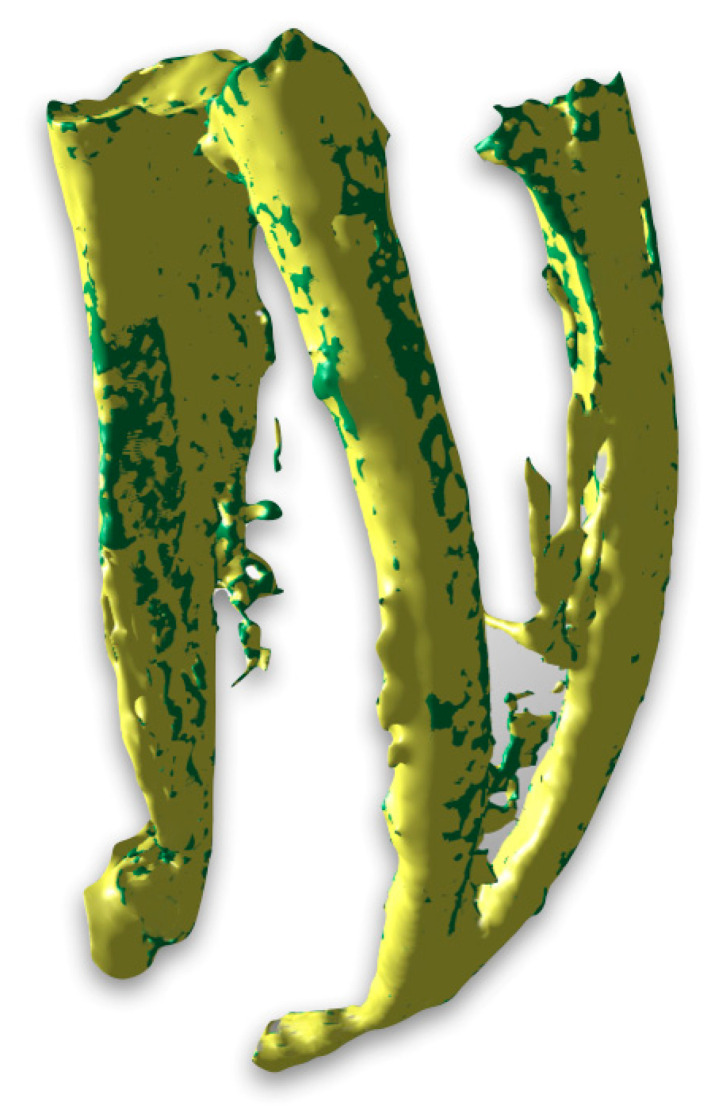
Representative µ-CT images of Group A (carrier-based obturation technique). Three-dimensional reconstruction was used to isolate the filling materials from the dental root system. This approach highlights the distinction between gutta-percha (displayed in green) and calcium-silicate-based sealer (displayed in yellow).

**Figure 4:**
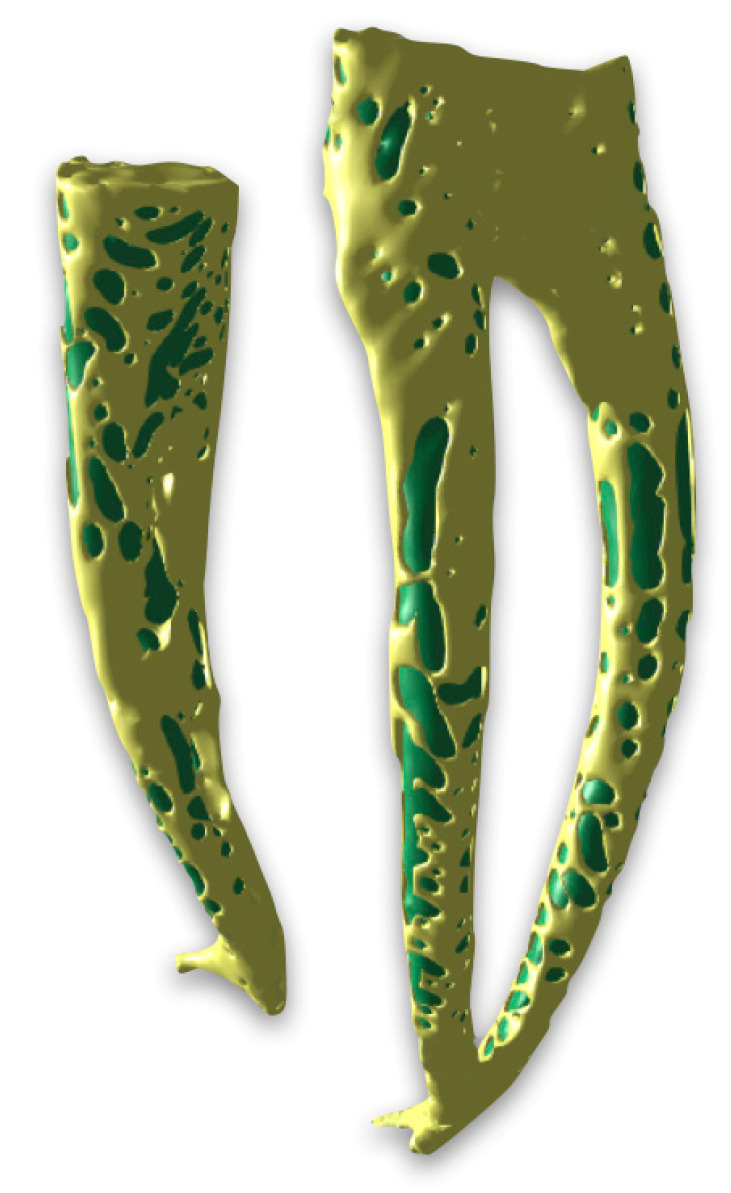
Representative µ-CT images of Group B (the single cone obturation technique). Three-dimensional reconstruction was used to isolate the filling materials from the dental root system. This approach highlights the distinction between gutta-percha (displayed in green) and calcium-silicate-based sealer (displayed in yellow).

The quantitative analysis focused on the overall number and total volume of voids per root canal, while the regional distribution of voids along the coronal, middle, and apical thirds was evaluated qualitatively using three-dimensional µ-CT reconstructions. Qualitatively, voids were detected in different areas of the root canal system, with distinct distribution patterns between the two groups. In Group A, voids were mainly observed in the coronal and middle thirds, while the apical third showed a more homogeneous filling pattern, as illustrated in Figure 1. In contrast, in Group B, voids were larger in size, with a greater total volume, and were predominantly concentrated in the coronal and middle thirds of the root canal. Additional voids were also observed in the apical third, indicating a less homogeneous filling along the entire root canal length (Figure 2). Nevertheless, in both groups, the apex appeared completely sealed without detectable voids. In addition, representative µ-CT images (Figure 3 and Figure 4) were used to further analyze the obturation techniques by differentiating the filling components. This analysis enabled a clear distinction between gutta-percha and CSS, allowing assessment of their spatial distribution within the root canal system and the localization of voids. The WCB method produced a significantly lower number and volume of voids, resulting in a denser and more homogeneous filling throughout the root canal system, as shown in Figure 3.

In contrast, the SC technique, while ensuring apical sealing, exhibited significantly larger and more frequent voids, predominantly in the coronal and middle thirds, as shown in Figure 4.

The quantitative analysis confirmed these observations, as reported in Table 1.

**Figure 5:**
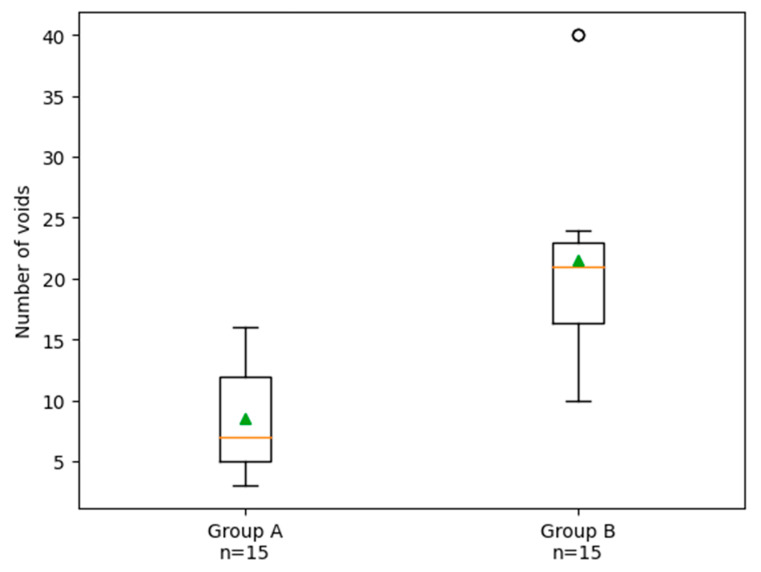
Box plot illustrating the distribution of the number of voids in Group A (carrier-based obturation) and Group B (single-cone obturation). The green triangle indicates the mean value, the horizontal line represents the median, and the box shows the interquartile range; whiskers indicate data dispersion. The empty circle indicates an outlier. Each data point represents one root canal system (one tooth). The sample size was n = 15 for each group.

Group A showed a significantly lower number of voids (*p* = 0.00002) and void volume (*p* = 0.0026) compared with Group B. In particular, the number of voids was significantly lower in Group A (9 ± 5.0) compared with Group B (22 ± 10.1, *p* = 0.0002), as reported in Figure 5.

**Figure 6:**
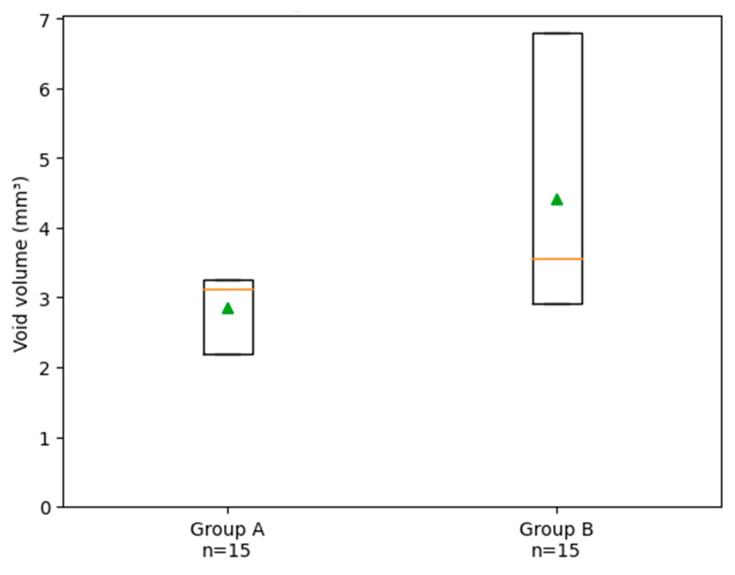
Box plot illustrating the distribution of void volume (mm^3^) in Group A (carrier-based obturation) and Group B (single-cone obturation). The green triangle indicates the mean value, the horizontal line represents the median, and the box shows the interquartile range; whiskers indicate data dispersion. The empty circle indicates an outlier. Each data point represents one root canal system (one tooth). The sample size was n = 15 for each group.

Similarly, the total void volume was significantly reduced in Group A (2.58 ± 0.8 mm^3^) compared to Group B (4.71 ± 1.1 mm^3^, *p* = 0.0026), as reported in Figure 6.

Overall, both the qualitative and quantitative analyses revealed statistically significant differences between the two obturation techniques (*p* < 0.001).

## 4. Discussion

A three-dimensional seal of the root canal system is essential for the long-term success of endodontic therapy. CSSs are increasingly used as biomimetic obturation materials, as their ion release and bioactivity promote a mineralizing seal within dentin and support a favorable biological response [29]. Nevertheless, the optimal obturation technique for achieving a predictable hermetic seal with these materials remains controversial. In recent years, the SC technique combined with CSS has regained popularity due to its simplicity, reduced operative time, and lower costs compared with warm obturation techniques [30,31]. However, WCB techniques are still widely regarded as the gold standard, as thermoplasticized gutta-percha can better adapt to canal irregularities and reduce interfacial gaps [32,33]. In the present study, both qualitative and quantitative µ-CT analyses revealed statistically significant differences between the two obturation techniques performed with the same CSS. Therefore, the null hypothesis was rejected, as the obturation technique significantly influenced three-dimensional filling quality when a CSS was used. Although both techniques achieved a consistent apical seal, significant differences were observed in void number and void volume, which were used as three-dimensional surrogate parameters to infer filling homogeneity, particularly in the middle and coronal thirds of the canal. Specifically, CSS tended to completely fill the apical region, where the canal lumen is narrower, resulting in an effective seal regardless of the obturation technique. In contrast, in the middle and coronal thirds, characterized by wider and more complex anatomy, CSS distribution was less homogeneous, and voids were more frequently detected. In these regions, the WCB technique provided a denser and more homogeneous filling compared with the SC approach, confirming that the obturation technique plays a relevant role in determining filling quality when CSS are employed. Beyond void detection, µ-CT enabled discrimination among gutta-percha and sealer components, allowing a detailed three-dimensional assessment of material distribution within the root canal system. The novelty of the present study lies in the methodological approach based on three-dimensional µ-CT analysis with digital segmentation of the obturation materials, which allowed CSS to be isolated from gutta-percha. This approach enabled a precise visualization and assessment of the spatial distribution and behavior of the sealer within the root canal system, independently of the obturation technique used. These findings highlight the limitations of sealer-dependent techniques in wider canal areas and underscore the importance of obturation methods capable of enhancing material compaction and adaptation. Consistent with previous reports, the SC technique has been shown to be less effective than warm obturation approaches, mainly due to limited adaptation of standardized gutta-percha cones to canal walls and inadequate filling of isthmuses and lateral canals [34,35,36]. Conversely, warm obturation techniques, including carrier-based and continuous-wave approaches, have been associated with denser fillings and reduced gap formation, as thermoplasticized gutta-percha can flow into canal irregularities and improve interfacial adaptation [37]. The use of warm obturation techniques in combination with CSS remains debated, primarily due to concerns derived from in vitro studies suggesting that elevated temperatures may interfere with setting kinetics, hydration reactions, and physicochemical stability [38]. Excessive heat may induce partial dehydration of the sealer, potentially affecting flowability, film thickness, and sealing ability [39]. However, such effects have largely been reported under experimental conditions that do not accurately reproduce the clinical thermal environment. Available evidence indicates that carrier-based obturation systems do not sustain high intraradicular temperatures, as thermal energy is rapidly dissipated following carrier insertion, resulting in temperature levels well below those reported to negatively influence CSS [40,41,42]. Consequently, thermal exposure during clinical warm obturation procedures appears transient and limited [42]. The present findings support the feasibility of combining WCB techniques with NeoSEALER Flo, as temperature-related alterations described in laboratory settings may be clinically negligible when appropriate protocols are applied. When considered alongside the favorable biological properties and bioactivity of CSS, these results suggest that warm obturation techniques can be safely employed with NeoSEALER Flo in routine clinical practice [17]. Moreover, the setting characteristics of CSS may have influenced the outcomes observed in the present study. As hydraulic materials, CSS undergo a moisture-dependent setting reaction that progresses over time, leading to dimensional stability and slight expansion [10]. In simplified obturation approaches such as the SC, where the sealer constitutes a substantial portion of the filling mass, these setting dynamics play a critical role in determining final filling quality. In wider or irregular canal regions, limited compaction may increase the likelihood of void formation during the setting phase. Conversely, in WCB, the use of thermoplasticized gutta-percha enhances material compaction and adaptation, reducing sealer thickness and consequently limiting the impact of sealer setting behavior on the final obturation outcome. This interaction between obturation technique and sealer setting dynamics is consistent with the present µ-CT findings, which demonstrated comparable apical sealing but improved filling homogeneity in the middle and coronal thirds when warm obturation was applied. Clinically, the present data indicate that the choice of obturation technique significantly influences filling quality when CSSs are used. While the apical region appears consistently well sealed, differences emerge in the middle and coronal thirds, where canal morphology is more complex and space-dependent. In such cases, WCB techniques may be preferable to enhance material compaction and adaptation, potentially improving long-term sealing performance. Conversely, the SC technique combined with CSS may represent a clinically acceptable and time-efficient option in teeth with narrow, minimally flared canals and relatively simple anatomy, where adequate adaptation of the gutta-percha cone and sealer can be achieved. This study has some limitations. Anatomical variability of the root canal system may have influenced obturation outcomes and limits generalizability. Although µ-CT provides accurate three-dimensional evaluation of voids and material distribution, it does not allow direct assessment of their clinical relevance. Accordingly, longitudinal clinical studies are needed to determine whether void characteristics influence long-term treatment success. Finally, the long-term behavior of CSSs exposed to thermal stress during warm obturation remains unclear, and future investigations should evaluate different formulations to clarify potential heat-related effects over time.

## 5. Conclusions

Within the limitations of this in vitro µ-CT study, the obturation technique significantly influenced 3D filling quality when a CSS was used. Although both techniques achieved a consistent apical seal, WCB resulted in a denser and more homogeneous filling, with fewer voids in the middle and coronal thirds compared with the SC. These findings highlight the influence of obturation technique on material distribution and void formation when CSSs are employed, particularly beyond the apical region. Further studies are needed to determine the clinical implications of these differences under varying anatomical conditions.

## Figures and Tables

**Table 1:** Comparison of the number and volume of voids between Group A (carrier-based obturation) and Group B (single-cone obturation). Values are expressed as mean ± standard deviation (SD).

Outcome	Group A	Group B	*p*-Value
Number of voids	9 ± 5.0	22 ± 10.1	0.00002 *
Void volume (mm^3^)	2.58 ± 0.8	4.71 ± 1.1	0.0026 *

* Statistically significant at *p* < 0.001.

## Data Availability

The original contributions presented in this study are included in the article. Further inquiries can be directed to the corresponding author.

## Supplementary Materials

The following supporting information can be downloaded at: https://www.mdpi.com/article/10.3390/biomimetics11020152/s1; Table S1: Volumetric classification of voids based on micro-CT resolution.

## References

[1] Ng Y.-L., Mann V., Gulabivala K. (2011). A Prospective Study of the Factors Affecting Outcomes of Nonsurgical Root Canal Treatment: Part 1: Periapical Health. Int. Endod. J..

[2] Iandolo A., Armogida N.G., Mancino D., Spagnuolo G., Cernera M., Abdellatif D. (2025). Evaluation of Root Canal Cleaning and Irrigant Penetration Using Different Irrigation Protocols: A Combined SEM and Single-Tooth Micro-CT Study. Clin. Exp. Dent. Res..

[3] (2006). European Society of Endodontology Quality Guidelines for Endodontic Treatment: Consensus Report of the European Society of Endodontology. Int. Endod. J..

[4] Wong A.W.-Y., Zhang S., Li S.K.-Y., Zhang C., Chu C.-H. (2017). Clinical Studies on Core-Carrier Obturation: A Systematic Review and Meta-Analysis. BMC Oral Health.

[5] Peng L., Ye L., Tan H., Zhou X. (2007). Outcome of Root Canal Obturation by Warm Gutta-Percha versus Cold Lateral Condensation: A Meta-Analysis. J. Endod..

[6] Buchanan L.S. (1994). The Continuous Wave of Condensation Technique: A Convergence of Conceptual and Procedural Advances in Obturation. Dent. Today.

[7] Zhang P., Yuan K., Jin Q., Zhao F., Huang Z. (2021). Presence of Voids after Three Obturation Techniques in Band-shaped Isthmuses: A Micro-computed Tomography Study. BMC Oral Health.

[8] Sauro S., Carvalho R.M., Ferracane J. (2025). The Rise of Advanced Bioactive Restorative Materials: Are They Redefining Operative Dentistry?. Dent. Mater..

[9] Camilleri J. (2020). Materials for Dentistry—Raising the Bar. Front. Dent. Med..

[10] Camilleri J., Atmeh A., Li X., Meschi N. (2022). Present Status and Future Directions: Hydraulic Materials for Endodontic Use. Int. Endod. J..

[11] Camilleri J. (2020). Classification of Hydraulic Cements Used in Dentistry. Front. Dent. Med..

[12] Guivarc’h M., Jeanneau C., Giraud T., Pommel L., About I., Azim A.A., Bukiet F. (2020). An International Survey on the Use of Calcium Silicate-Based Sealers in Non-Surgical Endodontic Treatment. Clin. Oral Investig..

[13] Modaresi J., Mokhtari F., Khodarahmi E. (2025). In Vitro Comparison of the Marginal Adaptation of Cold Ceramic Sealer with the Single-Cone Obturation Technique versus AH-26 Sealer with the Lateral Compaction Technique in Single-Canal Teeth. BMC Oral Health.

[14] Cardinali F., Camilleri J. (2023). A Critical Review of the Material Properties Guiding the Clinician’s Choice of Root Canal Sealers. Clin. Oral Investig..

[15] Debelian G., Trope M. (2016). The Use of Premixed Bioceramic Materials in Endodontics. G. Ital. Endod..

[16] Pontoriero D.I.K., Ferrari Cagidiaco E., Maccagnola V., Manfredini D., Ferrari M. (2023). Outcomes of Endodontic-Treated Teeth Obturated with Bioceramic Sealers in Combination with Warm Gutta-Percha Obturation Techniques: A Prospective Clinical Study. J. Clin. Med..

[17] Zamparini F., Spinelli A., Cardinali F., Ausiello P., Gandolfi M.G., Prati C. (2023). The Use of Premixed Calcium Silicate Bioceramic Sealer with Warm Carrier-Based Technique: A 2-Year Study for Patients Treated in a Master Program. J. Funct. Biomater..

[18] Patel F.H., Mehta T.K., Bhavsar K.A., Attur K.M., Attur S.K., Patel A.B. (2025). Enhancing Root Canal Filling Homogeneity: Investigating Cross Linked and Injection Molded Techniques against Single Cone Method. J. Conserv. Dent. Endod..

[19] Pirani C., Camilleri J. (2023). Effectiveness of Root Canal Filling Materials and Techniques for Treatment of Apical Periodontitis: A Systematic Review. Int. Endod. J..

[20] Sabeti M.A., Karimpourtalebi N., Shahravan A., Dianat O. (2024). Clinical and Radiographic Failure of Nonsurgical Endodontic Treatment and Retreatment Using Single-Cone Technique with Calcium Silicate-Based Sealers: A Systematic Review and Meta-Analysis. J. Endod..

[21] Olivieri J.G., Encinas M., Nathani T., Miró Q., Duran-Sindreu F. (2024). Outcome of Root Canal Retreatment Filled with Gutta-Percha Techniques: A Systematic Review and Meta-Analysis. J. Dent..

[22] Bardini G., Bellido M.M., Rossi-Fedele G., Casula L., Dettori C., Ideo F., Cotti E. (2025). A 4-Year Follow-up of Root Canal Obturation Using a Calcium Silicate-Based Sealer and a Zinc Oxide-Eugenol Sealer: A Randomized Clinical Trial. Int. Endod. J..

[23] World Medical Association World Medical Association (2013). Declaration of Helsinki: Ethical Principles for Medical Research Involving Human Subjects. JAMA.

[24] Tosco V., Monterubbianesi R., Aranguren J., Furlani M., Riberti N., Putignano A., Orsini G. (2025). Evaluation of Morphological and Chemical Composition of Dental Pulp Stones: A Combined Microanalytical Approach. J. Endod..

[25] Casino-Alegre A., Aranda-Verdú S., Zarzosa-López J., Rubio-Climent J., Plasencia-Alcina E., Pallarés-Sabater A. (2022). Intratubular Penetration Ability in the Canal Perimeter Using HiFlow Bioceramic Sealer with Warm Obturation Techniques and Single Cone. J. Clin. Exp. Dent..

[26] Tosco V., Monterubbianesi R., Aranguren J., Memè L., Putignano A., Orsini G. (2023). Evaluation of the Efficacy of Different Irrigation Systems on the Removal of Root Canal Smear Layer: A Scanning Electron Microscopic Study. Appl. Sci..

[27] Alkahtany S.M., AlHussain A.A., AlMthen H.A., AlDokhi H.D., Bukhary S.M., Almohaimede A.A., AlNeshmi B. (2024). Obturation Quality of Bioceramic Sealers with Different Obturation Techniques: A Micro-CT Evaluation. Sci. Rep..

[28] Bender D., Ocak M., Uzunoğlu Özyürek E. (2024). Root Canal Cleanliness and Debris Extrusion Following Retreatment of Thermoplastic Injection Technique and Bioceramic-Based Root Canal Sealer. Clin. Oral Investig..

[29] Saber S., Raafat S., Elashiry M., El-Banna A., Schäfer E. (2023). Effect of Different Sealers on the Cytocompatibility and Osteogenic Potential of Human Periodontal Ligament Stem Cells: An In Vitro Study. J. Clin. Med..

[30] Bunashi E.S., Hu M., Lee A.H.C., Zhang C., Kishen A., Chang J.W.W. (2025). Enhancing the Quality of Single Cone Obturation Using Hydroxyapatite Precursor Grafted Nanocomplex for Dentine Conditioning: An In Vitro Study. Int. Endod. J..

[31] Zhang Q., Meng X., Zhan J., Huo L., Lei Y. (2025). Sealing Ability of the Single-Cone Obturation Technique with Bioceramic Sealer iRoot SP in Oval Root Canals: An in Vitro Study. BMC Oral Health.

[32] Gok T., Gok A., Aciksoz H.O. (2025). Assessment of Gap Areas of Root Filling Techniques in Teeth with 3D-Printed Different Configurations of C-Shaped Root Canals: A Micro-Computed Tomography Study. BMC Oral Health.

[33] Vazquez-Alcaraz S., Gancedo-Caravia L., Arias A., Bascones J. (2024). Performance of Obturation Techniques in Anatomical Irregularities Located at Different Thirds of the Root Canal System. J. Appl. Oral Sci..

[34] Nagarajan V., Ahamed A.S., Sreekrishnapillai B., Rajaraman G., Elangovan S.K., Guptha R.V. (2024). Comparative Evaluation of Volume and Homogeneity of Obturation with Four Different Obturation Systems Using Micro-Computed Tomography: An In Vitro Study. J. Pharm. Bioallied Sci..

[35] Alim B.A., Garip Berker Y. (2020). Evaluation of Different Root Canal Filling Techniques in Severely Curved Canals by Micro-Computed Tomography. Saudi Dent. J..

[36] Zhao Y., Zhang Y., Shi J., Zheng G., Chen Y., Zou D., Pan Y. (2025). Evaluation of Two Instrumentation Techniques and Obturation Methods in Mandibular First Premolar C-Shaped Canals by Micro-CT. BMC Oral Health.

[37] Sadat S.M.A.E., Chew H.P., Fok A., Elashiry M.M., ElShenawy A.M., Saber S. (2024). Quality of Different Obturation Techniques to Fill Perforating Internal Root Resorption: A Micro-Computed Tomographic Study. BMC Oral Health.

[38] Scardini I.L., Masiero A.V., Dos Santos M., Teixeira F.B. (2025). Influence of Heating on the Physicochemical Properties of Novel Calcium Silicate-Based Endodontic Sealers. J. Endod..

[39] Camilleri J. (2015). Sealers and Warm Gutta-Percha Obturation Techniques. J. Endod..

[40] Janini A.C.P., Moraes B.F., Pelepenko L.E., Dos Santos V.A.B., Barros-Costa M., Malosá G.F., de Souza Batista F.R., de Aguiar Silveira Meira J., Matsumoto M.A., Antunes T.B.M. (2025). Physicochemical Properties and Biological Interaction of Calcium Silicate-Based Sealers—In Vivo Model. Clin. Oral Investig..

[41] Kooanantkul C., Shelton R.M., Camilleri J. (2023). Comparison of Obturation Quality in Natural and Replica Teeth Root-Filled Using Different Sealers and Techniques. Clin. Oral Investig..

[42] Donnermeyer D., Schäfer E., Bürklein S. (2018). Real-Time Intracanal Temperature Measurement During Different Obturation Techniques. J. Endod..

