# Supplementary material for: Single-Cone vs. Carrier-Based Root Canal Obturation with a Calcium-Silicate-Based Sealer: An In Vitro µ-CT Analysis
Source: Biomimetics (Basel). 2026 Feb 19;11(2):152. doi: 10.3390/biomimetics11020152 (PMC12937792; doi:10.3390/biomimetics11020152)
Supplement: Supplementary file 1 [file biomimetics-11-00152-s001.zip › biomimetics-4138544-supplementary.pdf]

Supplementary Table S1. Volumetric classification of voids based on micro-CT resolution

**Void category**	**Volume range (mm^3)**	**Rationale**
Below detection threshold	$< 4.1 \times 10^{-5}$	Volumes below the minimum detectable void volume (MDVV), corresponding to objects smaller than 27 voxels (3 × 3 × 3), excluded to minimize image noise
Small voids	$\geq 4.1 \times 10^{-5}$ and $< 1.0 \times 10^{-3}$	Voids with limited volumetric extent, slightly above the MDVV and reliably detectable by the micro-CT system
Medium voids	$\geq 1.0 \times 10^{-3}$ and $< 1.0 \times 10^{-2}$	Voids with intermediate volumetric extent
Large voids	$\geq 1.0 \times 10^{-2}$	Voids with large volumetric extent and greater spatial relevance

The MDVV was calculated based on a voxel size of 11.5 µm, assuming a minimum cluster of 27 voxels.
